# 746 Routinely Collected Burn Clinical Data in Canada: Determining the Knowledge Gap

**DOI:** 10.1093/jbcr/irae036.289

**Published:** 2024-04-17

**Authors:** Sabrina Wang, Eduardo I Gus, Claudia C Malic, Jennifer Zuccaro

**Affiliations:** University of Toronto, Toronto, ON; SickKids / University of Toronto, Toronto, ON; CHEO, Ottawa, ON; Hospital for Sick Children, Toronto, ON; University of Toronto, Toronto, ON; SickKids / University of Toronto, Toronto, ON; CHEO, Ottawa, ON; Hospital for Sick Children, Toronto, ON; University of Toronto, Toronto, ON; SickKids / University of Toronto, Toronto, ON; CHEO, Ottawa, ON; Hospital for Sick Children, Toronto, ON; University of Toronto, Toronto, ON; SickKids / University of Toronto, Toronto, ON; CHEO, Ottawa, ON; Hospital for Sick Children, Toronto, ON

## Abstract

**Introduction:**

Unlike other developed countries that hold national burn registries to monitor burn injury and care, Canada relies on single-centre secondary datasets and administrative databases as surveillance mechanisms. The objective of this study is to determine the knowledge gap faced in Canada for not having a dedicated burn registry.

**Methods:**

A comprehensive scoping review was conducted to identify the burn literature that has arisen from secondary datasets in Canada. Literature of all study designs was included with the exception of case reports and cases series. Once data extraction was concluded, a thematic framework was constructed based on the information that arose from nations that hold national burn registries.

**Results:**

Eighty-eight studies were included. Twelve studies arose from national datasets, and 18 from provincial databases, most of which were from Ontario and British Columbia. Only seven studies were conducted using a combination of Canadian units’ single-centre datasets. The majority of included studies (58%) resulted from non-collaborative use of single-centre secondary datasets. Research efforts were predominantly conducted by burn units in Ontario, British Columbia, Manitoba and Alberta. A significant number of the included studies were outdated and there were several provinces/territories with no published burn data whatsoever.

**Conclusions:**

Efforts should be made towards the development of systems to surveil burn injury and care in Canada. This study supports the development of a working group and potentially a nation-wide burn registry to bridge this knowledge gap.

**Applicability of Research to Practice:**

Decisions regarding burn injury prevention and delivery of burn care must be informed by clinical data, in order to maximize efficacy and outreach. Canada has a publicly funded healthcare system, and yet, these decisions at present are not informed by data. The establishment of the knowledge gap that is faced by the Canadian burn community is the first step towards the establishment of a working group that will discuss how Canada will move forward to bridge this gap and increase the quality of burn care delivered across the country.

